# An open automation system for predatory journal detection

**DOI:** 10.1038/s41598-023-30176-z

**Published:** 2023-02-20

**Authors:** Li-Xian Chen, Shih-Wen Su, Chia-Hung Liao, Kai-Sin Wong, Shyan-Ming Yuan

**Affiliations:** 1 School of Big Data, Fuzhou University of International Studies and Trade, Fuzhou, 350202 China; 2 Department of Computer Science, National Yang Ming Chiao Tung University, Room 702, MIRC, No.1001, University Road, Hsinchu, 30010 Taiwan

**Keywords:** Environmental social sciences, Engineering, Mathematics and computing

## Abstract

The growing number of online open-access journals promotes academic exchanges, but the prevalence of predatory journals is undermining the scholarly reporting process. Data collection, feature extraction, and model prediction are common steps in tools designed to distinguish between legitimate and predatory academic journals and publisher websites. The authors include them in their proposed academic journal predatory checking (AJPC) system based on machine learning methods. The AJPC data collection process extracts 833 blacklists and 1213 whitelists information from websites to be used for identifying words and phrases that might indicate the presence of predatory journals. Feature extraction is used to identify words and terms that help detect predatory websites, and the system’s prediction stage uses eight classification algorithms to distinguish between potentially predatory and legitimate journals. We found that enhancing the classification efficiency of the bag of words model and TF-IDF algorithm with diff scores (a measure of differences in specific word frequencies between journals) can assist in identifying predatory journal feature words. Results from performance tests suggest that our system works as well as or better than those currently being used to identify suspect publishers and publications. The open system only provides reference results rather than absolute opinions and accepts user inquiries and feedback to update the system and optimize performance.

## Introduction

Predatory journals are considered a significant threat to the trustworthiness and legitimacy of mainstream scientific research and reporting^1,2^. Defined as deceptive or write-only publications^3,4^, predatory journals and predatory conference proceedings cater to the growing demand among scholars to have their research published^5–8^. They promote themselves as having rapid manuscript review processes but often fail to mention that they do not adhere to standard peer-review procedures. Some predatory journals are known for using false information to lure researchers into submitting manuscripts and then demand exorbitant article processing charges (APCs) prior to publication^1,9,10^. As of 2021, Cabells’ Predatory Reports database shows that there were approximately 15,000 active predatory journals, with authors collectively paying hundreds of thousands of dollars to have their papers published^11^.

When inaccurate or poorly executed research results are published in predatory journals, they can affect subsequent studies and the veracity of information disseminated to the general public^1^. In some cases, these and other specious research results get posted on websites or media outlets such as Facebook, Twitter, and Line or reported by local TV and radio stations^12^. In many cases, individuals who read or hear these stories are not given sufficient information to verify the original sources, eventually creating a situation where news consumers cannot distinguish among three types of science: legitimate, junk, and pseudo^13^. A simple example comes from Taiwan, where a research team claimed to have found data indicating that eating pineapple fruit every day was a sufficient alternative to medical treatments for eye spots known as vitreous floaters^14^. When the original article was published in 2019, all of Taiwan’s major news channels reported its findings, which were reposted on several social media websites. However, researchers who reviewed the study in detail found multiple points to challenge, such as the complete absence of participant demographic data, the lack of a control group, incorrect statistical methods, and a combination of exaggeration and basic grammar errors throughout the written report. In a second example from the *Macedonian Journal of Medical Science*, a group of researchers claimed that “there may be a black hole-like structure at the center of the earth.” The same author of this report wrote a paper claiming that coronaviruses are caused by 5G network radiation^15^. Experts who re-examined these studies speculated that the authors deliberately submitted ridiculous manuscripts to a predatory journal or that an artificial intelligence program was used to insert critical terms and phrases into a paper to make it look like legitimate research.

The past two decades have witnessed a dramatic increase in the number of open access (OA) journals. Since the purpose of scientific and professional journals is to convey information in ways that allow for verification and replication by other researchers in shared communities^16^, OA journals can serve an important role in terms of information mobility and dissemination, especially since many OA publications are free or inexpensive, and allow for the rapid online distribution of the latest findings^7,9,17^. However, the ease of online publishing has resulted in explosive growth in the number of online journals, which presents challenges in terms of determining the quality of published research^8^. Since universities and research institutes require scholars to publish in journals with high rankings in SCI, SSCI, or other indexes, there is a particular concern for identifying and avoiding predatory and low-quality journals^6^.

Predatory journals succeed because they cater to the demands of academic promotions and tenure, annual scholarly assessments, and job applicant appraisals, with evaluations based on numbers of publications, author order, and journal impact^5–7^. Today the “publish or perish” adage is especially true in developing countries such as China, India, and South Africa, with scientists facing tremendous pressure to have their research cited^7,18^. Certain geographic areas are recognized for their participation in the publication process—for example, the highly developed eastern coastal cities of China. Researchers in certain countries (e.g., India, Nigeria, Turkey) have reputations for the enormous numbers of papers published in predatory journals^6^. Academic communities in other countries are taking steps to resist this phenomenon. The Center for Taiwan Academic Research Ethics Education is working with National Yang Ming Chiao Tung University, National Taiwan University, and other schools and research institutes to help faculty members and researchers learn how to identify predatory journals and conferences. Training involves practice in three steps: thinking, checking, and submitting to legitimate journals and publishers. Participants are also shown how to use library resources such as Beall’s List, the Stop Predatory Journals List, the Directory of Open Access Journals (DOAJ), the Master Journals List, the International Network for the Availability of Scientific Publications, and African Journals Online. However, there is no comprehensive one-stop inquiry system for use by scholars interested in publishing their manuscripts.

Jeffrey Beall, credited with coining the term “predatory open access publishing”^9,19^, describes the OA business model as charging exceptionally high processing fees for manuscripts to be published in “free” online journals^9^. His heuristic criteria for identifying predatory journal websites include article acceptance on topics unrelated to the journal’s stated field, promises of rapid review and publication, and the charging of exceptionally high APCs without guarantees of reasonable editorial services^9,20^.

Researchers currently use a combination of systematic reviews and statistical analyses when examining journal lists and websites. Both quantitative (frequencies and percentages of predatory journal characteristics) and qualitative methods (thematic analyses) were applied to detect predatory journal markers in five commonly used bibliographic databases^21^. They reported that in 78% of the studies they reviewed for their project, the authors used comments, opinions, letters, or editorials to delineate or discuss journal characteristics. They extracted 109 unique characteristics from the remaining 22% and used them to establish six analytical categories: journal operations; article, editorial, and peer review procedures; communication; article processing charges; dissemination, indexing, and archiving; and the appearance of five descriptors. Their findings highlight a long list of yellow flags: deceptive practices or lack of transparency indicating poor quality standards, marks of unethical research or publishing practices, the use of certain kinds of persuasive language, journals published by authors for limited audiences in specifically identified countries, unclear information about APCs, and claims of being listed in well-known indexes or databases, among others. Other bibliometric analysis to check international medical literature on predatory publishing^22^ and predatory journal citers^23^. The descriptive bibliometric methods were used to analyze the productivity of individuals, institutions, and nations and the citing authors’ geographic location and publications.

In a separate paper, manual classification methods tend to rely on inadequate or inherently confusing criteria^6^. Thus, there is a small number of identification programs that use different approaches from that described by Cobey et al.^21^. One example, a web-based plug-in from ispredatory.com, uses a combination of Beall’s list and predatory publisher data shared by scholars—a form of crowdsourcing^24^. Users can search for publishers by name, URL, title, or journal ISSN and access a manually updated list of confirmed predatory publishers. Data pattern extraction strategy for false indexing claim detection uses elements of random forest, RepTree, and J48 decision trees plus related algorithms^25^. To determine links between individual articles and predatory/legitimate publishers and journals, a data-driven training model, called PredCheck, was used with datasets from two India-based publishing groups: OMICS (OPG) and BioMedical Central (BMC)^26^. On average, their naive Bayes classifier-based model attained 95% accuracy and an F1 score of 0.89.

These methods and criteria use the specific file, tag content, or web plug-in and therefore require human intervention to gather information for verification purposes. For scholars, there is currently no open query system that does not require installation and is more intuitive. The present study aims to design an intuitive analytical system that anyone can use without a plug-in to identify predatory journals and publisher websites. Our proposed solution involves model training using predatory and legitimate datasets constructed from journal website content. Our proposed system, which uses the smallest possible number of features to achieve its purpose, makes use of diff scores (to be described in a later section) to identify feature words commonly found on predatory journal websites—in other words, terms that exert positive impacts on model performance. We have created a web application that provides full public access to the AJPC system. This is an easy-to-use query system for reference.

## Related research

### Predatory and legitimate journals

Predatory journals take advantage of scholars' eagerness to submit papers to solicit articles. Features include quick review without a professional review mechanism, fraudulent impact factor, fake editorial boards truthlessly listing respected scientists, an extensive collection of articles, journal titles seemingly similar to those of legitimate journals, and aggressive spam invitations to submit articles. Furthermore, predatory journals make profits by charging high article processing fees.

**Figure 1:**
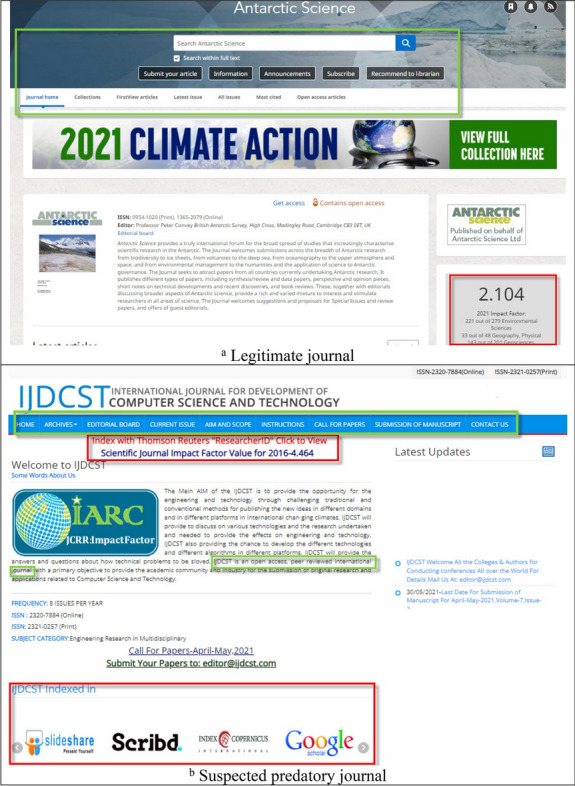
Our proposed academic journal predatory checking (AJPC) system identified the first journal, *Antarctic Science*, as legitimate, and the second, *International Journal for Development of Computer Science and Technology*, as potentially predatory. Similarities between the two websites are noted in the color box frames 1a was captured from https://www.cambridge.org/core/journals/antarctic-science# and 1b was captured from http://ijdcst.com/.

As shown in Fig. 1, both predatory and legitimate journal websites commonly display text blocks labeled “Impact factor,” “Editorial board,” “About the journal,” and “Contact us.” Distinguishing between them requires the same machine learning tactics used to resolve binary classification problems such as fake social media identities^27^, suspicious URLs in social networks, and the hijacking of legitimate websites^25^. In machine learning, the text classification process consists of tag or category assignments based on text content. Although text can offer rich sources of information, extracting insights can be difficult and time-consuming when unstructured data are involved.

Tactics used by predatory publishers include misrepresentations of peer review processes, editorial services, and database indexing statuses^1^. Profit-oriented predatory journals generally cut back drastically on editorial and publishing costs by completely eliminating procedures such as referee reviews, addressing academic misconduct issues, flagging possible instances of plagiarism, and confirming author group legitimacy^29^. Nevertheless, a surprising number of predatory journals find it easy to attract scholarly submissions from authors interested in padding their CVs^21,30^. These purposefully deceptive actions can result in incorrect quotes and citations, thus wasting precious research funds and resources while destroying public confidence in university research. Predatory journal websites also tend to lack credible database indexing with agencies such as Journal Citation Reports (JCR) or the Directory of Open Access Journals (DOAJ). Combined, these problems are creating chaos in academic communities, with editors, authors, reviewers, and related individuals pursuing various strategies to protect research quality^31,32^.

Since predatory journals tend to falsify their index information and impact values while promoting high acceptance rates^33^, researchers interested in avoiding predatory journals must be familiar with current index rankings, scientific indicators, and announcements from science publication databases. Along with editorial office addresses, phrases and terms such as “indexing in [specific] database” and “journal metrics” appear to indicate legitimacy, but they are also used in misleading advertising and promotional emails sent out by predatory journals^34^. Other red flags include promises of fast peer review; the use of informal or personal contact emails that are not associated with a website; journal webpages with multiple spelling, grammar, and content errors; false claims of high impact factors with self-created indicators; and lack of publisher listings in universal databases such as the DOAJ, the Open Access Scholarly Publishers Association, or Committee on Publication Ethics^13,19,33–36^. Unintentionally publishing academic research through spam and phishing emails may damage careers and loss of money caused. Researchers are troubled by the electronic invitations they receive to submit papers or attend conferences, and they need a good education or a valuable evaluation system to assess whether they are predatory or not.

**Figure 2:**
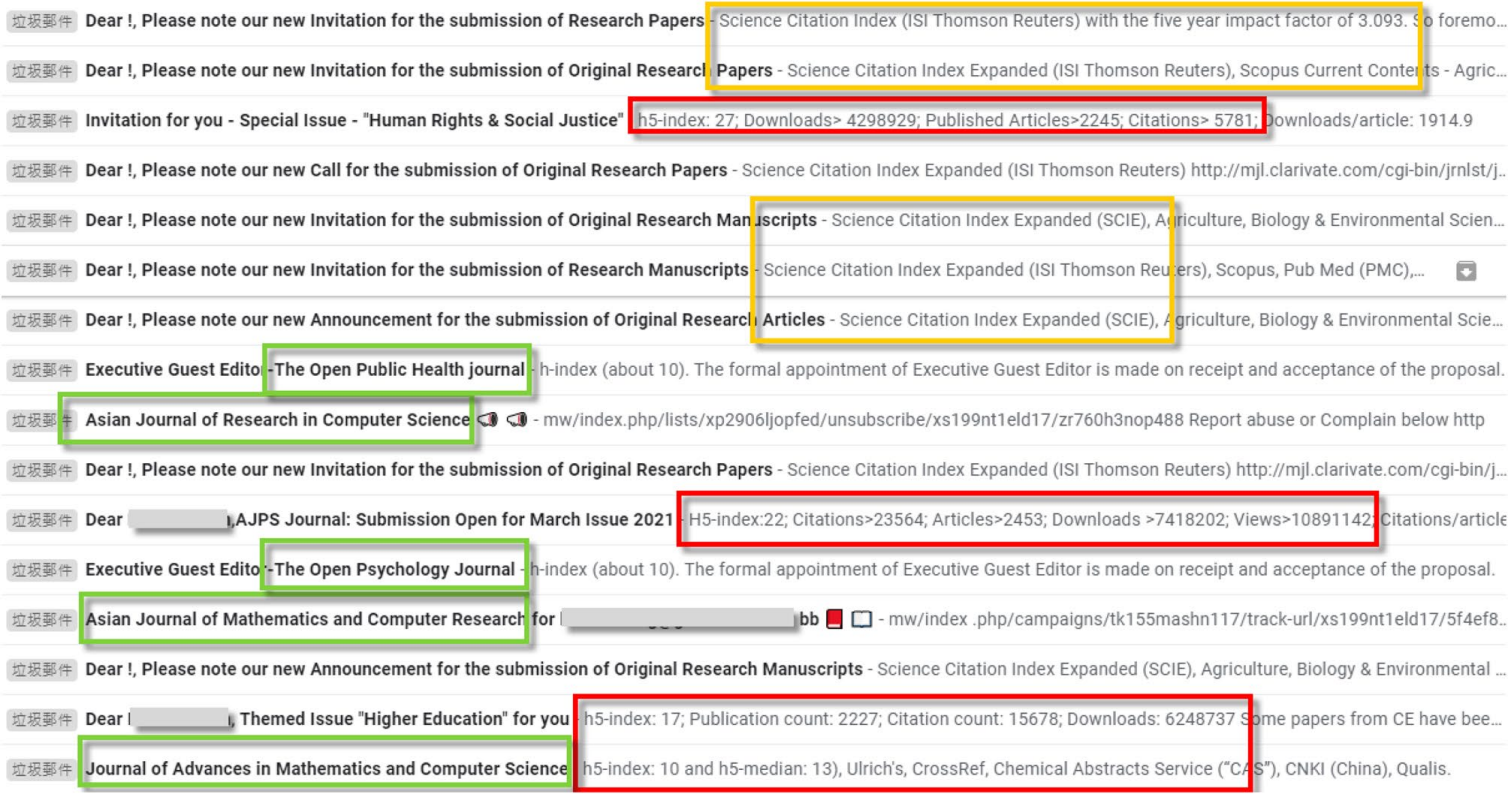
Examples of potentially misleading text in invitations sent to scholars to submit manuscripts.

As Fig. 1 shows, the owners of predatory journal websites are skilled at mimicking the layout styles of legitimate websites. Figure 2 shows the opening lines of letters and emails from predatory journals that scholars regularly receive inviting them to submit manuscripts; it is difficult to distinguish them from communications sent out by legitimate journals^21,30^. Both figures contain examples of text extolling the virtues of the inviting journals, including high h5-index values; high citation rates; and specific indexing (green, red and orange boxes, respectively).

### Classification model

Supervised, unsupervised, and reinforcement machine learning for natural language processing are useful tools for solving numerous text analytics problems. The primary challenge for creating a convenient predatory journal identification system is similar to those for fake news and malicious URL detection problems^28,37^: both problem types involve text variation, confusing or unclear messages, and imitative website layouts. Since predatory journal homepage identification is essentially a classification problem, we set out to modify one or more algorithms to improve the human-centered machine learning process associated with the Google UX Community^38^. Currently, the most commonly used text evaluation and classification approaches are support vector machine (SVM), Gaussian naïve Bayes, multinomial naïve Bayes, random forest (RF), logistic regression, stochastic gradient descent (SGD), K-nearest neighbor (KNN), and voting^39^. All use finely tuned parameters to select the best configuration for each classification technique. The following are brief descriptions of these approaches.

Frequently used to detect deceptive text, clickbait, and phishing websites, SVMs are practical tools that use decision planes to classify objects according to two categories: expected and non-expected^37,40,41^. An example of an SVM-based approach exploits content-based features to train classifiers that are then used to tag different categories (F1 = 0.93)^40^. Their SVM algorithm used each data set as a vector, plotted it in a high-dimensional space, and constructed a hyperplane to separate classes. The hyperplane maximized distances between planes and their nearest clickbait and non-clickbait data points.

The RF and two naïve Bayesian (NB) systems are frequently applied to text classification problems due to their computational efficiency and implementation performance^42^. However, the lack of algorithm-specific parameters means that NB system users must have a thorough knowledge of the model being examined, which adds a considerable computational burden for optimization purposes^43^. The RF system works as a random hyperlink with specific parameters—for instance, specific tree and variable numbers for each split. As long as the overall input size is sufficiently large, its performance is considered suitably robust to handle parameter changes. In a study designed to detect instances of phishing, the RF classifier had a 98.8% accuracy rate^41^, and in a separate study aimed at detecting predatory biomedical journals, it produced an F1 score of 0.93^26^. The RF system has also been used with decision trees as a strategy for preventing the indexing of papers published in predatory journals since some individuals have become skilled at hijacking journal websites and collecting processing and publication fees from unwary authors^25^.

Logistic regressions have been used to classify news headlines and content. In one study involving fake and true news stories in Bulgaria, a logistic regression approach achieved 0.75 accuracy for the most difficult dataset^44^. Logistic regressions assign weight factors to features in individual samples, with predicted results equal to each sample feature value multiplied by its impact factor—the equation coefficient. Accordingly, classification problems are transformed into optimization coefficient-solving problems.

*SGD* has been successfully applied to large-scale and sparse machine learning problems frequently encountered in text classification and natural language processing. It can be used for either classification or regression calculation purposes. In an Indonesian study, an SGD classifier with a modified huber kernel was used to detect hoaxes on news websites and was reported as having an 86% accuracy rate^35^.

KNN is an instance-based or lazy learning method, with local approximations and with all computations deferred until post-classification^45^. Considered one of the simplest of all machine learning algorithms, KNN is sensitive to local data structures. This method can be used with a training set to classify journals by identifying the closest groups. Category labels are assigned according to the dominance of a particular category within a group. One study applied heuristic feature representations with the KNN method to classify predatory journals, and reported a 93% accuracy rate^46^.

Voting is one of the easiest ways to combine predictions from multiple machine learning algorithms. The method does not entail an actual classifier, but a set of wrappers trained and evaluated in parallel to take advantage of each algorithm’s characteristics.

Classification entails two primary objectives: analyzing factors that affect data classification, and assigning elements to pre-established classes via feature predictions^39^. When a classifier has sufficient data, a model can identify the features of expected categories and use them for further data category predictions. For text classification purposes, if word order relationships and grammar structures in a file are not considered, a common vectorization method is *bag of words* (BOW), which calculates weights associated with the numbers of word occurrences in a text. BOW has frequently been applied to tasks involving restaurant review classification, negative information retrieval, and spam mail filtration^28,37,47^. To make use of machine learning algorithms, individual documents must be transformed into vector representations. Assuming N documents with T terms are used in all of them, it is possible to convert all documents into a vector matrix. For example, assume a vector N_3_ = [15, 0, 1,…, 3] with word T_1_ appearing 15 times, word T_3_ one time, and word T_t_ 3 times in document 3. Although BOW is considered a simple method for document transformation, two problems must be resolved, the first being that the total number of words per individual document is not the same. If there are 10,000 total words in document 2 and 50 in document N, and word 3 appears ten times in document 2 but only two times in document N, obviously it will have much greater weight in document N. The other problem is that idiomatic expressions and frequently used words exert significant impacts on individual documents. For instance, if a common word such as “the” appears many times in different documents but has the most appearances in one, it becomes a dominant but meaningless vector.

*Frequency-inverse document frequency* (TF-IDF) is a statistical method commonly used in information retrieval and text-related scenarios to evaluate word importance in documents^43,49,50^. The TF-IDF algorithm divides feature words in terms of weight and reduces the number of zero-weight words. For the predatory journal website problem, finding better feature word weights can improve discrimination efficiency if words can be identified as appearing more frequently in predatory websites. A short list of feature words that have been identified as possibly meeting this requirement includes “international,” “American,” “British,” “European,” “universal,” and “global,” with some researchers suggesting that they are more likely to appear in predatory journal titles^21,34,51^. Other suspect words are associated with metrics: “quality impact factor,” “global impact factor,” and “scientific journal impact factor” are three examples. Other feature words refer to ideas expressed in an earlier section of this paper: promises of peer review processes and short review cycles ranging from a few days to less than four weeks.

### Measuring the prediction performance of classification algorithms

Since early website pattern detection is central to identifying predatory journals, determining model accuracy is a critical task. Four performance metrics have generally been used to evaluate classifiers: accuracy (percentage of correct classification predictions), precision (proportion of correct positive identifications), recall (percentage of relevant documents successfully retrieved), and F1 score (average of precision and recall as a balanced index). For this study, we used recall and F1 scores as measures of classifier performance. F1 scores can be used to confirm recall and precision levels, with higher scores indicating fewer legitimate journal classification errors. Calculation methods for accuracy, precision, recall, and F1 scores are shown in Table 1.

**Table 1:** Definitions for the four performance metrics used for model evaluation.

Metric	Definition
Accuracy	$\frac{TP+TN}{TP+FP+TN+FN}$
Precision	$\frac{TP}{TP+FP}$
Recall	$\frac{TP}{TP+FN}$
F1-score	$$\frac{2*Precision*Recall}{Precision+Recall}$$

### System design

**Figure 3:**
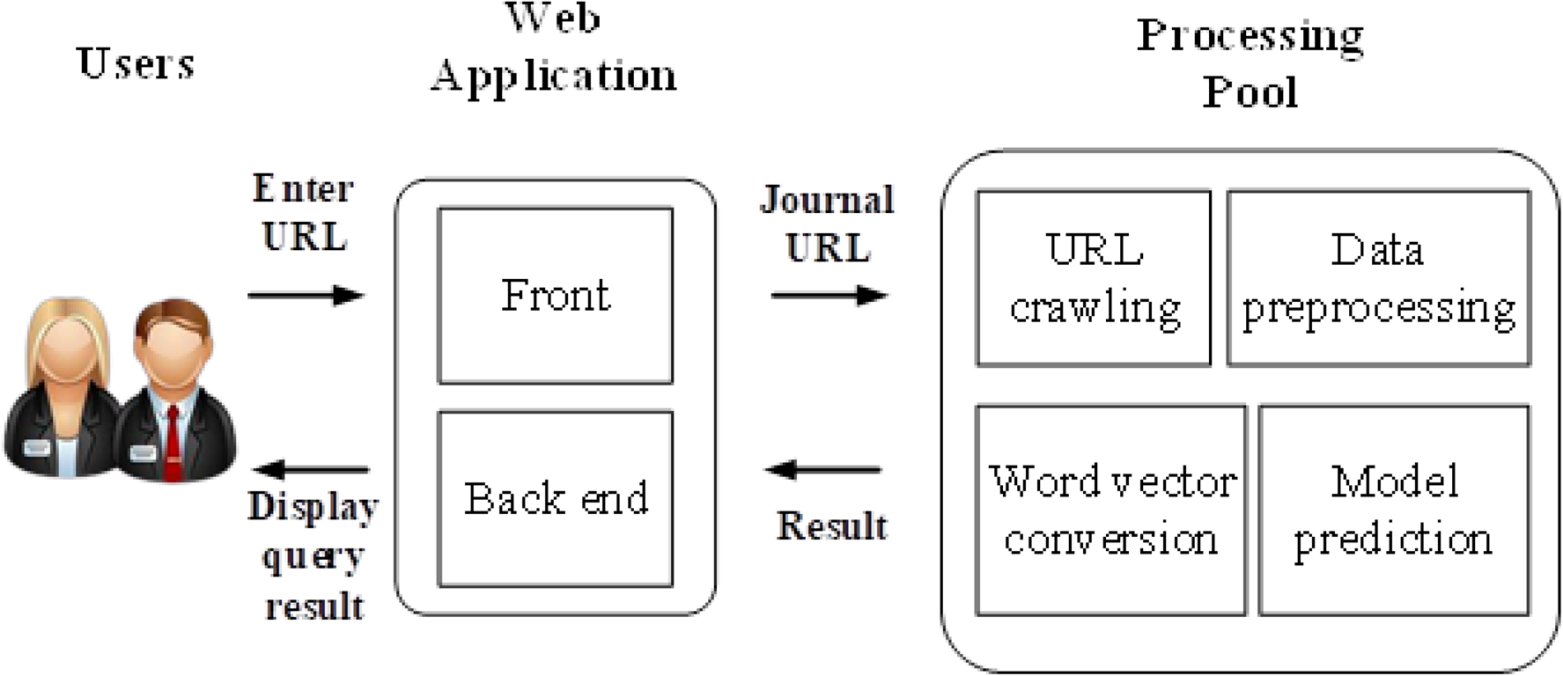
Proposed academic journal predatory checking (AJPC) system architecture.

Figure 3 presents the AJPC system architecture, constructed using Flask, a web application framework written in Python. AJPC extracts URL content entered by a user, preprocesses the data, converts website content into word vectors, and applies a classification model for category prediction before sending results to its back end and displaying them. In brief, AJPC consists of three main modules: data collection, feature extraction, and model prediction. Data collection during natural language preprocessing focuses on URL content for feature extraction using the BOW method. During the model prediction stage, eight common classifiers are applied to model training, with the best model selected based on recall rate and F1-score.

### Data collection

A single predatory journal list was established using information collected from updated Beall’s^19^ and the Stop Predatory Journals list^52^. Journals appearing on these lists are screened in terms of credibility as established by the Committee on Publication Ethics, long-term observations, and anonymous community-based feedback^19,52^. Legitimate journal list data were collected from the Berlin Institute of Health (BIH) Quest website^53^, which utilizes data from the DOAJ and Pubmed Central lists of journals. After manually checking all predatory and legitimate journal links to confirm active statuses, a web crawler was applied to create two lists. For this study AJPC identified 833 links to predatory journals and 1,213 to legitimate journals. In supervised machine learning, samples are normally divided into separate training and testing sets, with the first used to train the model and the second used to examine the performance of the model selected as the best.

**Figure 4:**
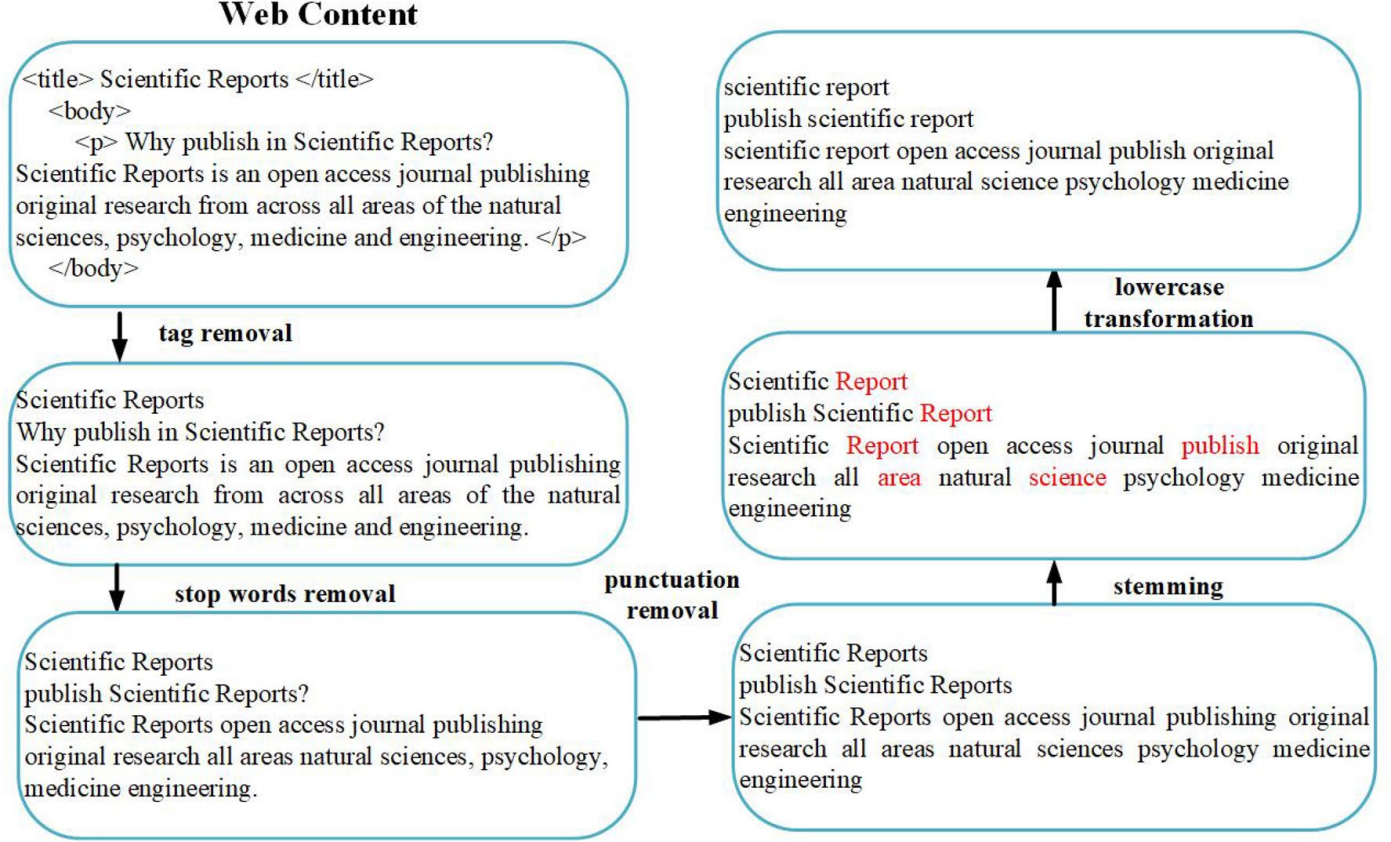
AJPC system preprocessing steps.

Data collection preprocessing procedures commonly entail the removal of tags, stop words, and punctuation, and the transformation of stems and lower case text^54^. In addition to reducing feature space dimensionality, these procedures promote text classification system efficiency^54,55^. In the example shown as Fig. 4, unnecessary tags (HTML, CSS) and scripts are filtered out, and some of the most commonly used “stop words” are removed—for example, “will” and “and” in the sentence, “Information Sciences will publish original, innovative, creative and refereed research articles.” “Publish,” “published” and “publishing” are examples of stem word variants; AJPC retains the stem word “publish” but removes the other two^56^. All text is converted to lower case to reduce the potential for different treatment for words using mixed upper- and lower-case letters.

### Feature extraction and data classification

The feature extraction module uses the BOW method, an efficient information retrieval tool for text data^19,57^. BOW converts text into numerical values and vectors that machine learning algorithms can process and use as input. As an example we will use two sentences:

“It was the best time for epidemic control,” (sentence 1)

“It was the time for economic recovery.” (sentence 2)

BOW records all occurrences of words in both sentences in a dictionary of the training corpus. This method looks up the dictionary when the sentence is converted to a vector. If the word in the sentence appears in the dictionary, the vector value is stored as 1; otherwise, it is stored as 0. For example, “time” is stored as 1 in each vector, and sentence 2’s words (i.e., “best,” “epidemic,” and “control”) are not in the dictionary and are stored as 0. In this example the two binary vectors are represented as [1, 1, 1, 1, 1, 1, 1, 1, 0, 0] and [1, 1, 1, 0, 1, 1, 0, 0, 1, 1]. These vectors are used to create two word sets, one associated with predatory journal websites and the other with legitimate websites. The TF-IDF method uses the sets to evaluate the degree of importance for individual words in a collection of documents. TF-IDF is believed to resolve two problems associated with the BOW algorithm: dealing with differences in total numbers of words in two or more articles, and recurring idiomatic words and expressions that exert significant influence in documents. As explained in an earlier example, if word ${w}_{2}$ appears nine times in document ${D}_{2}$ and two times in document ${D}_{t}$, but ${D}_{2}$ has 10,000 words and ${D}_{t}$ only 50 words, ${w}_{2}$ is much more important to file ${D}_{t}$.

TF refers to the frequency of a given word. With ${tf}_{t,d}$ expressed as

1 $${tf}_{t,d}= \frac{{q}_{t,d}}{{\sum }_{k}{q}_{k,d}},$$

where ${q}_{t,d}$ denotes the number of times that word t appears in document $d$ and ${\sum }_{k}{q}_{k,d}$ denotes the total number of words in document $d$. In other words, the TF method considers the importance of each word in terms of frequency rather than total number of appearances, with the most common words preprocessed by IDF. ${idf}_{t}$ denotes a word importance measure, expressed as

2 $${idf}_{t}=\mathrm{log}\left(\frac{D}{{d}_{t}}\right),$$

where D is the total number of words and ${d}_{t}$ is the number of documents containing word t. ${d}_{t}$ is larger and ${idf}_{t}$ smaller for words appearing in many articles. The value of word t in document d is calculated using a combination of TF and IDF, expressed as

3 $${score}_{t,d}= {tf}_{t,d} \times {idf}_{t}.$$

The value of ${score}_{t,d}$ is higher when word t appears more frequently in document d (i.e., a larger ${tf}_{t,d}$) and when it appears infrequently in other documents (i.e., a larger ${idf}_{t}$). Thus, if a predatory journal website contains “this,” “journal,” “is” and “international” and a legitimate journal website contains “this,” “journal,” “has,” “peer review,” and “step”, then the two websites are said to contain a total of 9 words. On the predatory journal website (d = 1), the score_2,1_ assigned to the word “journal” is $1/4*\mathrm{log}(9/1)$, and on the legitimate journal website (d = 2) the score_2,2_ assigned to the same word is $1/5*\mathrm{log}(9/1)$.

After building predatory and legitimate journal website datasets for TF-IDF score calculations, *diff* scores were used to identify feature words. A *diff* score representing the different appearances of word t in documents 1 (predatory) and 2 (legitimate) is calculated as

4 $${diff}_{t}= {score}_{t,1}- {score}_{t,2}.$$

Using the above example, ${diff}_{2}= 1/4*\mathrm{log}(9/1)-1/5*\mathrm{log}(9/1)$.

In this case, a larger *diff* value indicates that word t appears more often on predatory than on legitimate journal websites, therefore it may have greater utility for identifying the predatory or legitimate status of a website. The rankings of individual words based on their *diff* scores were used to create a feature word set consisting of n words. Table 2 lists the 20 feature words that appeared most frequently on the predatory journal websites used in this study.

**Table 2:** Top 20 feature words identified by the proposed academic journal predatory checking (AJPC) system.

Rank	Feature	Rank	Feature
1	Journal	11	Biochemistry
2	Issue	12	Engineering
3	International	13	Index
4	Volume	14	Review
5	Paper	15	Doi *
6	Research	16	Biology
7	Science	17	Molecular
8	Factor	18	Peer
9	Impact	19	Submission
10	Publication	20	Field

*Doi (digital object identifier) is a term designating an intellectual copyright for a name or idea posted on the Internet.

The text content of all 833 predatory and 1,213 legitimate journal websites was converted into vectors. Specifically, a 1 × n vector was constructed for each website, with vector t set to 1 when word t was one of the top n feature words in journal j_i_, and to 0 if word t did not appear as a top feature word. For example, if the top 5 feature words were identified as “journal,” “issue,” “international,” “volume” and “paper,” and the journal j_i_ text content includes “journal,” “research,” “international,” “information” and “paper,” the resulting j_i_ word vector used for model training and prediction was [1, 0, 1, 0, 1]. The primary goal of classification is to determine categories or classes for new data. Classification can be performed with either structured or unstructured data. Each classifier requires parameter optimization to achieve the most accurate results. Following data collection and feature extraction, 80% of the journals in our sample (666 predatory, 970 legitimate) were randomly selected for use as a training set; the remaining 20% (167 predatory, 243 legitimate) was used as a testing set. Model training also utilized the top 50–9,000 feature words.

## Results

### AJPC system

**Figure 5:**
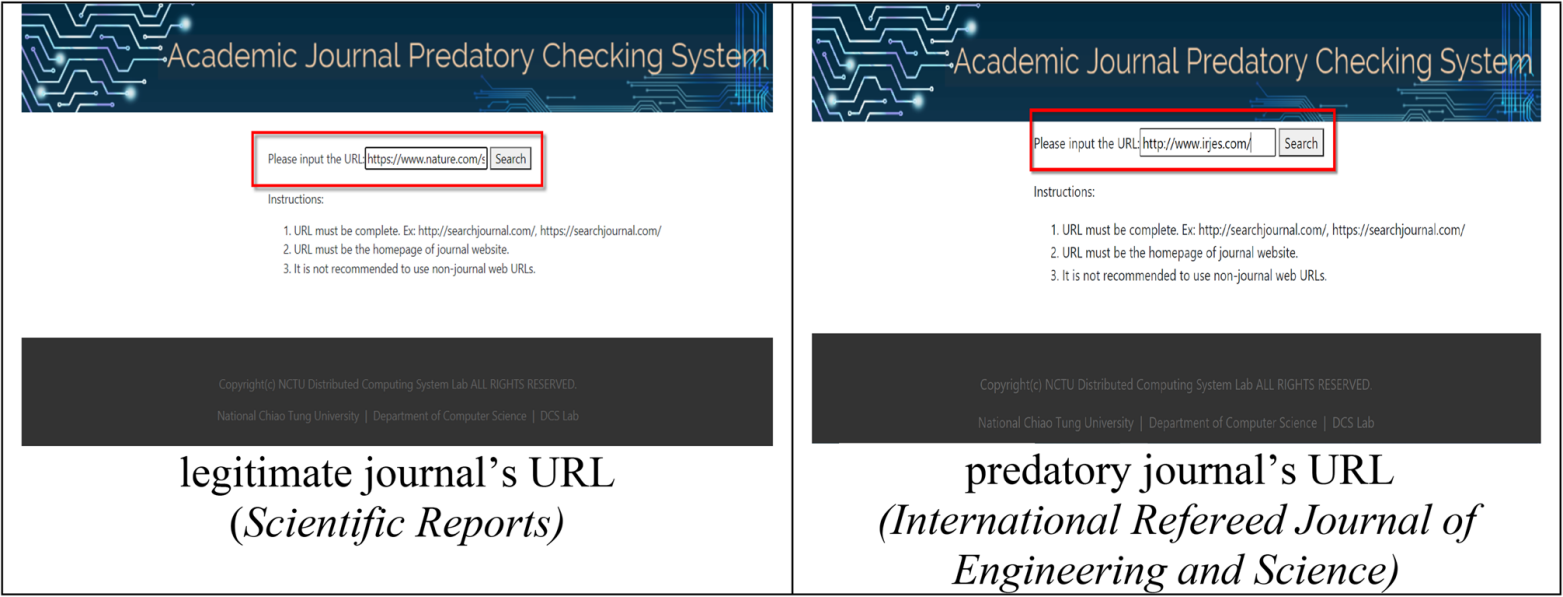
Legitimate and predatory journal query examples.

**Figure 6:**
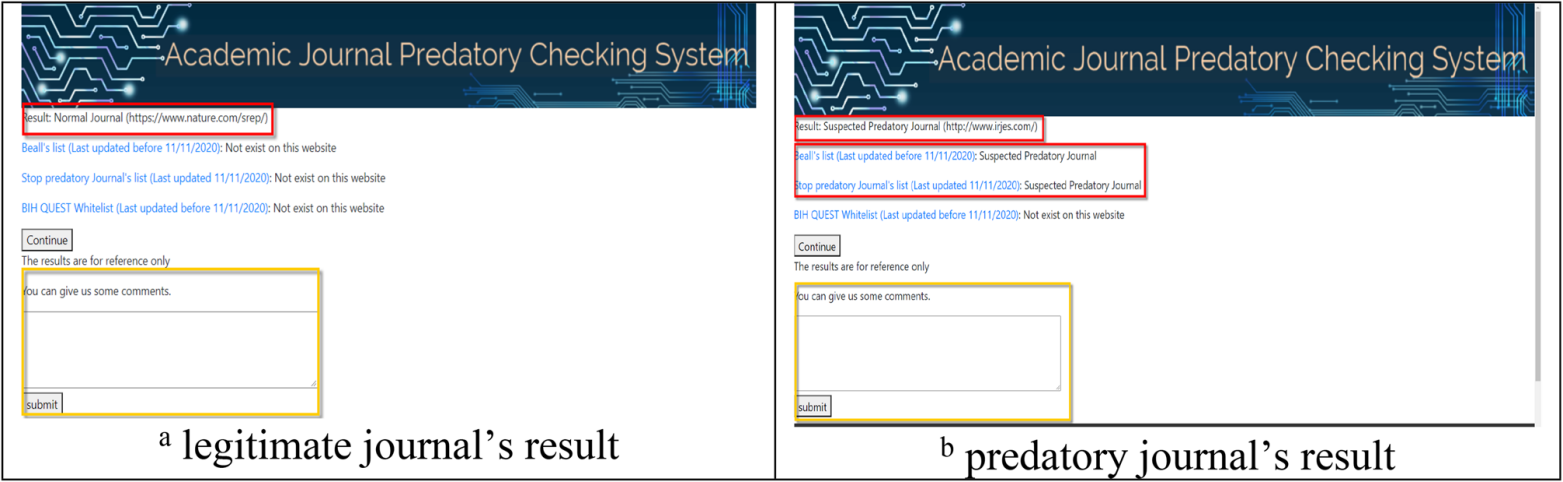
Legitimate and predatory journal query results returned by the AJPC system.

**Figure 7:**
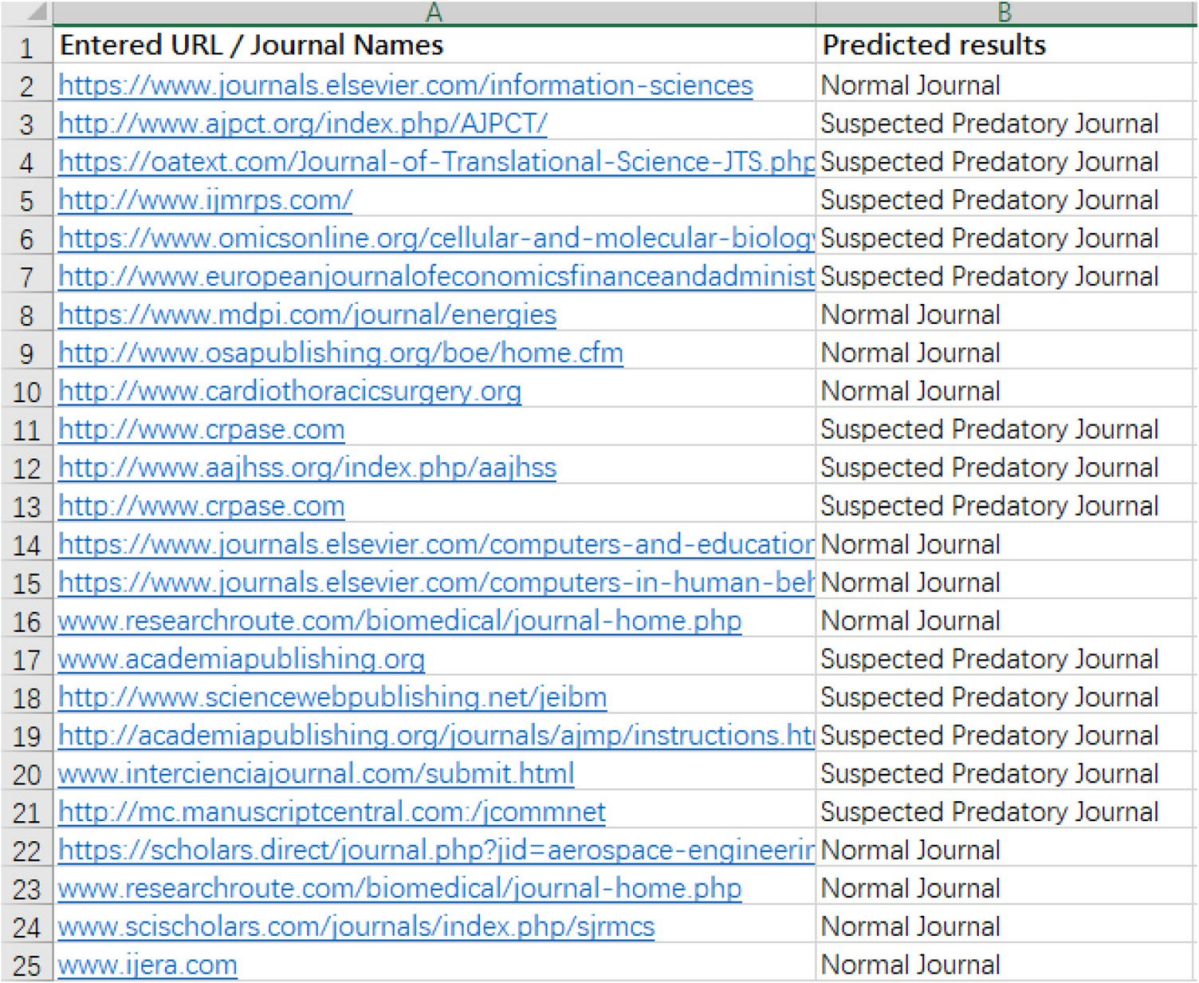
Legitimate and predatory journals’ queried results returned by AJPC system.

In the web version of the AJPC system, user queries (journal website URL or name) are sent to the preprocessing tool (Fig. 5). After performing all of the above-described operations, the website gives a “normal” message for legitimate journals and a “does not exist on this website” message for journals that do not appear on the Stop Predatory Journals, updated Beall’s or BIH QUEST lists. All other results trigger a “suspected predatory journal” message. Examples of AJPC query results are shown in Figs. 6a,b. Users can contribute additional recommendations to optimize model performance to strengthen system classification capabilities. As shown in Fig. 7, the website back end collects all user query results for additional system model training. The AJPC system’s classification evaluation methods were detailed in the following section.

The eight classification algorithms were examined to identify the most useful one for predatory journal identification tasks, and to identify feature words that may be useful for distinguishing between legitimate and predatory journal websites. The parameter settings to train the classifiers were shown as Table 3. Our examination was conducted using Python 3.7.3 with a server running the Ubuntu 18.04 operating system (Intel Core i5-8400 CPU @ 2.80 GHz processor, NVIDIA GTX-1600 GPU, 16 GB RAM). Our experiment goals were to (a) identify the best model results in terms of accuracy, precision, recall rate and F1 score, and (b) determine whether predatory journal websites could be classified correctly (i.e., the model classification efficiency is better when the recall rate is higher), and whether the individual models did not classify legitimate journals as predatory (i.e., F1 scores close to 1 were viewed as indicators of model success.) The following is a summary of our recall rate and F1 score results.

**Figure 8:**
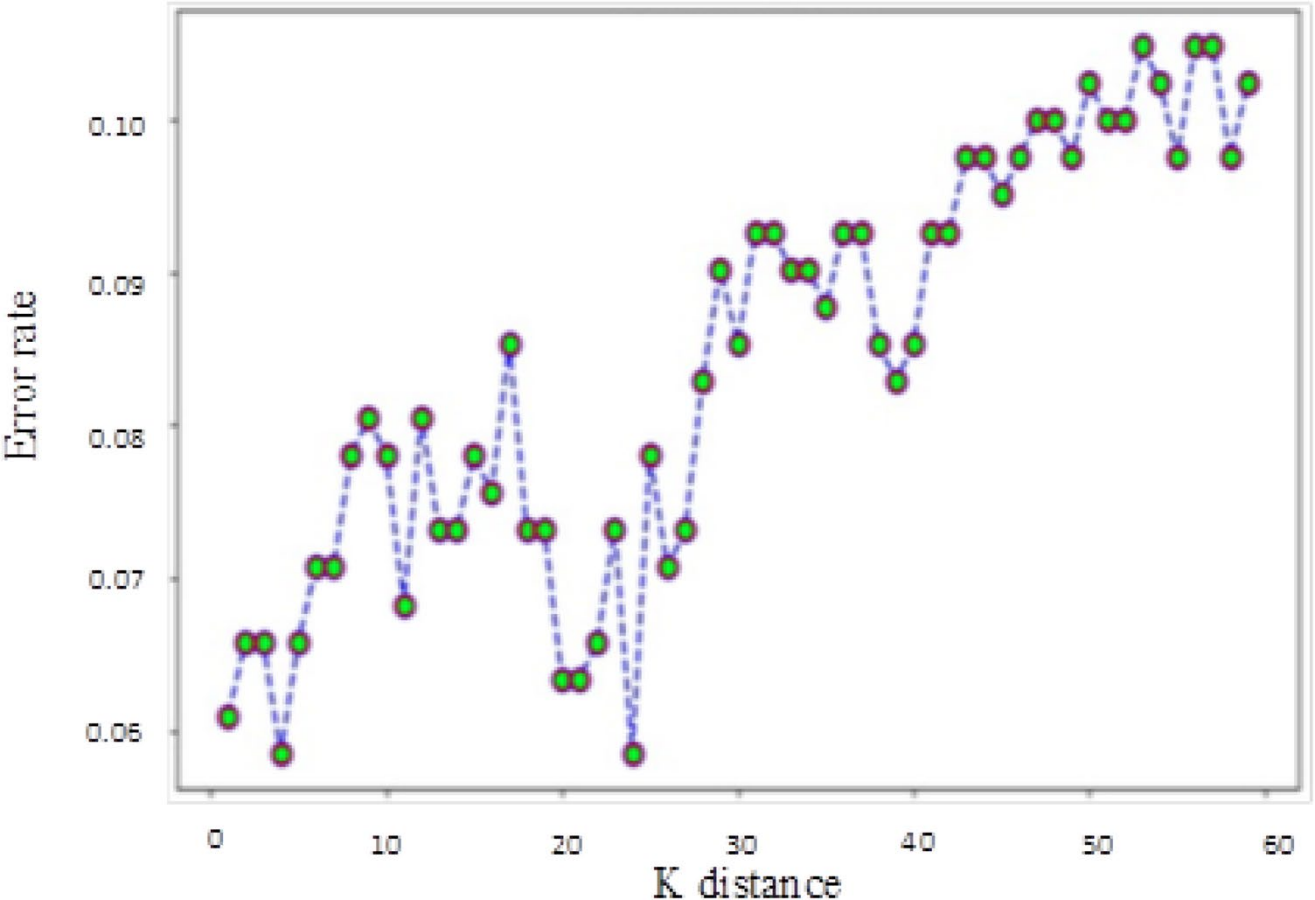
Relationship between K distance and error rate in KNN classifier algorithm.

**Figure 9:**
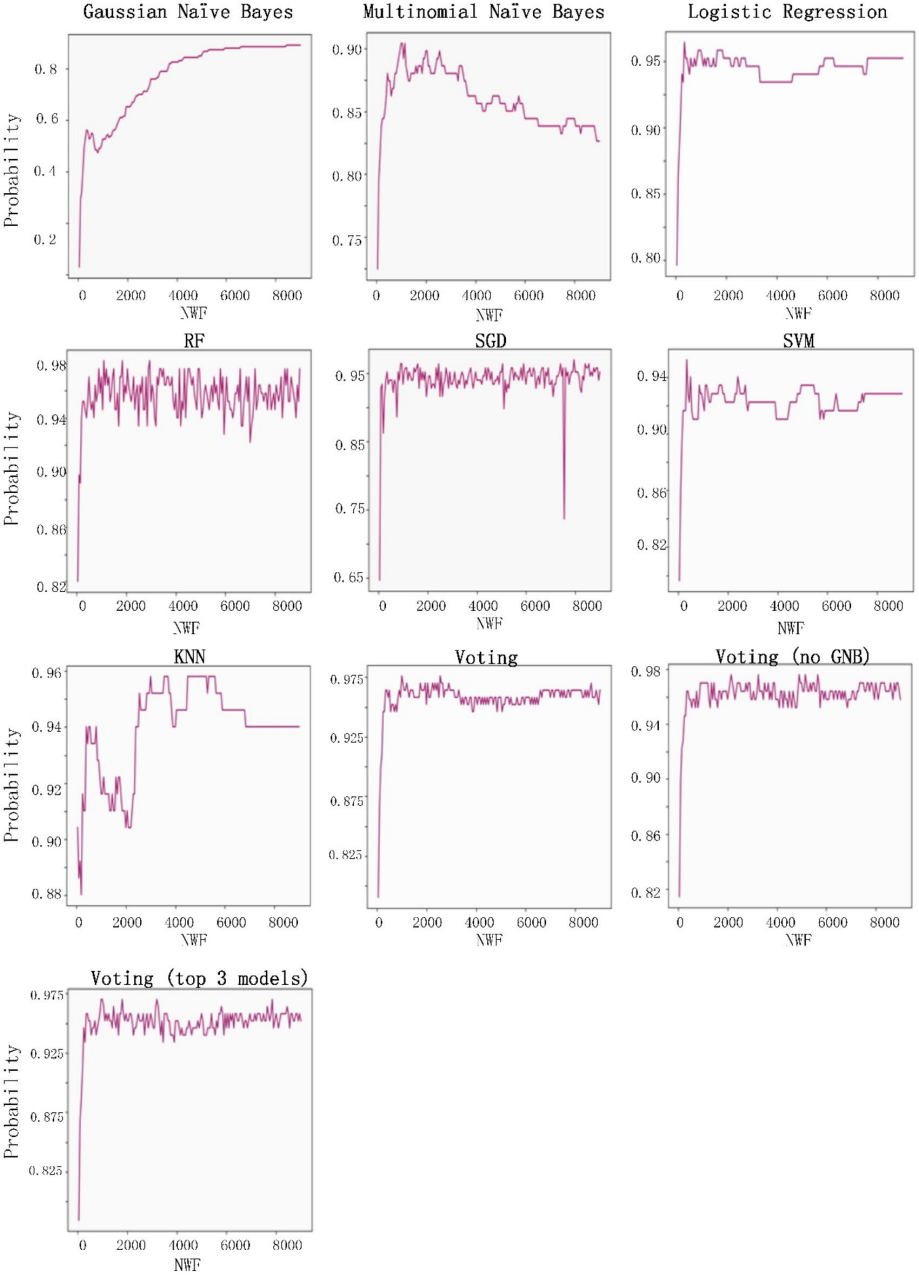
Recall rate performance data for the eight classifiers examined in this study.

**Figure 10:**
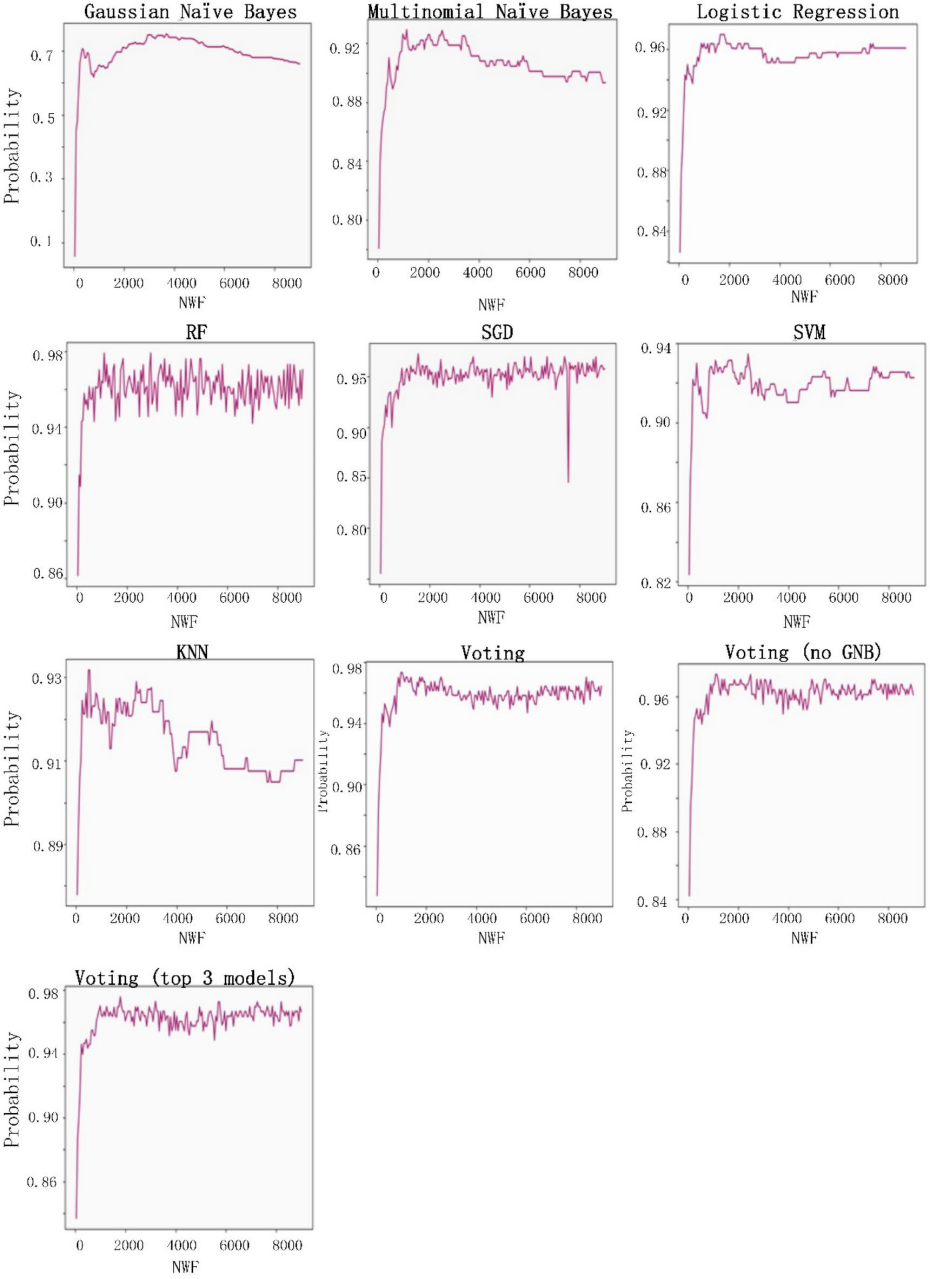
F1-score performance data for the eight classifiers examined in this study.

1. A Gaussian naïve Bayes (GNB) algorithm can be applied to multiple variable types when predatory features conform to Gaussian distributions^45^. During the model prediction step, we observed a recall rate of 0.89 when the number of word features (NWF) was 8,450, and an F1 score of 0.752 when NWF = 3,700.
2. A multinomial naïve Bayes (MNB) algorithm is suitable for discrete feature classifications^58^. Multinomial distributions usually require integer feature counts, but fractional counts such as those used with TF-IDF can also work. The MNB method is primarily used with document classification problems, especially those involving word frequency. Our experiment results indicate a maximum recall rate of 0.904 when NWF = 1,000 and an F1 score of 0.93 when NWF = 1,150.
3. Logistic regressions are supervised learning algorithms primarily used to solve binary classification problems^59^. When generating logistic regression equations, maximum likelihood ratios are applied to determine the statistical significance of variables. One characteristic of logistic regressions is that all returned values range between 0 and 1. By determining whether a value is greater or less than 0.5, data can be classified using a 0 or 1 label. Our regression results indicate a maximum recall of 0.964 when NWF = 350 and an F1 score of 0.97 when NWF = 1,650.
4. Random forest (RF) ensemble learning algorithms combine several models to produce a single stable and robust model free from bias and overfitting^60^. Random forests are thought of as a combination of multiple decision trees, with each tree producing a separate prediction. RF “votes” are generated by training data bootstrap samples and random feature selection. Predictions receiving the most votes are selected as final, with category tags determined by the best results for individual decision trees. These algorithms randomly select multiple features to identify the best parameters at all decision tree nodes. This selection process works well in situations consisting of multiple features per vector, since it mitigates interdependence among feature attributes. Our prediction results indicated an RF recall rate of 0.982 when NWF = 850 and an F1 score of 0.98 when NWF = 1,200.
5. *SGD algorithms* represent a simplified method for finding local function minimums^61^. One advantage of SGD algorithms is the possibility of obtaining models with loss values within acceptable ranges without the requirement of sample extraction. However, there is a potential for noise triggered by samples that cannot move in optimal directions during all iterations. Prediction results for SGD indicate a maximum recall rate of 0.97 when NWF = 7,950 and an F1 score of 0.972 when NWF = 1,550.
6. *SVM algorithms* are known for their classification performance with multidimensional and non-linear data^62^. These algorithms use statistical risk minimization to estimate classified hyperplanes. The primary purpose of an SVM algorithm is to locate maximum decision boundaries between distinguishable labels. For example, when weight and refractometer data are used to distinguish between an orange and a tangerine, their values are respectively set along the x-axis and y-axis, resulting in a classification line separating the two. Our SVM model prediction results indicate a maximum recall rate of 0.952 when NWF = 350, and an F1 score of 0.934 when NWF = 2,400.
7. KNN classification algorithms are effective tools for problem domains with unknown densities^45,63^. After calculating distances between targeted data and individual data points, a KNN algorithm uses the minimum data distance K to calculate the number of tags to which each data point belongs before predicting the maximum number of labels for the targeted data.
  However, this method sometimes leads to overfitting when K = 1. If K is equal to the number of training examples, then the number of predicted results equals the maximum number of labels. For this reason, the KNN classification algorithm calculated the K error rate (i.e., error rate = error classified count / total test set size) and observed a minimum rate of 0.065 when K = 4 (Fig. 8); the category parameter for the KNN neighbor was therefore set to 4. The KNN prediction results indicate a maximum recall rate of 0.96 when NWF = 3,000 and a maximum F1-score of 0.93 when NWF = 500.
8. The voting method combines the above seven classification algorithms^64^. Each algorithm is given a predatory/legitimate “vote,” and the result receiving the most votes is selected. Our data from voting predictions indicate a recall rate of 0.97 when NWF = 2,900 and an F1 score of 0.973 when NWF = 1700. After removing the poorly performing Gaussian naïve Bayes algorithm from the voting list, the highest recall rate was 0.976 when NWF = 2,150 and the highest F1 score 0.97 when NWF = 1,100. We then used the three highest recall model results (random forest, SGD and logistic regression) to determine predatory labels. A maximum recall of 0.97 occurred when NWF = 950 and a maximum F1 score of 0.975 was observed when NWF = 1,800. In other words, the results for these three models were almost identical. Combined experimental prediction results are shown in Table 4 and Figs. 9 and 10.

**Table 3:** Parameter settings to train the classifiers.

Model	Parameters
GNB	priors = None
MNB	alpha = 1.0
Logistic Regression	solver = 'liblinear'
Random Forest	n_estimators = 50, random_state = 1
SGD	penalty = ”l2”
SVM	kernel = 'linear', probability = True
KNN	n_neighbors = 4
Voting	estimators = [('gnb', clf1), ('mnb', clf2), ('svm', clf3), ('sgd', clf4), ('lr', clf5), ('rf', clf6), ('knn', clf7)], voting = 'hard'
Voting (no GNB)	estimators = [('mnb', clf2), ('svm', clf3), ('sgd', clf4), ('lr', clf5), ('rf', clf6),('knn', clf7)], voting = 'hard'
Voting (the top 3 model)	estimators = [('sgd', clf4), ('lr', clf5), ('rf', clf6)],voting = 'hard'

Abbreviations: GNB, Gaussian naïve Bayes; MNB, multinomial naïve Bayes; SGD, stochastic gradient descent; SVM, support vector machine; KNN, K-nearest neighbor.

clf1 = GNB, clf2 = MNB, clf3 = SVM, clf4 = SGD, clf5 = LogisticRegression, clf6 = RandomForest, clf7 = KNeighborsClassifier.

**Table 4:** Prediction results for individual classifier algorithms.

Model	Number of Word Features (NWF)	Recall	Number of Word Features (NWF)	F1
GNB	8450	0.89	3700	0.752
MNB	1000	0.904	1150	0.93
Logistic Regression	350	0.964	1650	0.97
Random Forest	850	0.982	1200	0.98
SGD	7950	0.97	1550	0.972
SVM	350	0.952	2400	0.934
KNN	3000	0.945	500	0.931
Voting	2900	0.97	1700	0.973
Voting (no GNB)	2150	0.976	1100	0.972
Voting (the top 3 model)	950	0.94	1800	0.975

GNB, Gaussian naïve Bayes; MNB, multinomial naïve Bayes; SGD, stochastic gradient descent; SVM, support vector machine; KNN, K-nearest neighbor.

We checked the false-negative results for each classification model in an effort to confirm predatory/legitimate website classification accuracy. For the 167 websites in the test data set, the random forest model had the best performance in terms of both prediction (0.982 recall rate, 0.98 F1 score) and classification accuracy (false-negative = 2). We therefore selected this model for use with the AJPC back end. Misclassification data are shown in Table 5.

**Table 5:** False-negative numbers for all classifiers.

Model	False-negative	Model	False-negative
GNB	18	SVM	6
MNB	16	KNN	7
Logistic Regression	6	Voting	5
Random Forest	**2**	Voting (no GNB)	4
SGD	5	Voting (the top 3 model)	5

GNB, Gaussian naïve Bayes; MNB, multinomial naïve Bayes; SGD, stochastic gradient descent; SVM, support vector machine; KNN, K-nearest neighbor.

## Discussions

In machine learning, it is generally assumed that the more pronounced the characteristics of classifier training, the better the results produced by a classification model. Unlike the text-based classification methods described in Bedmutha et al.^26^ and Adnan et al.^46^, our proposed system uses *diff* scores (a measure of differences in specific word frequencies between journals) to identify feature word sets for classification prediction purposes. AJPC also provides objective data from three predatory journal lists: updated Beall’s, Stop Predatory Journals, among others. As Moussa^65^ noted that retracting a published article from a predatory journal is almost impossible. In many cases, the inability to distinguish legitimate journals from the increasing amount of spam emails can deprive valuable research manuscripts of the chance to be published in better journals. Further, the proposed system reduces the potential for predatory journal publishers to harming scholarly value, and provides suggestions for submitting articles.

If the predatory journals were judged as legitimate journals, it caused serious consequences. If the recall rate is higher, it means that the rate of legitimate journals being predicted correctly is higher. In addition, maintaining a high F1 score does not sacrifice too much precision. Bedmutha et al.^26^ used research articles to training the model and got more than 0.71 F1 score in engineering area and 0.9 F1 score in biomedical area. Adnan et al.^46^ utilized Heuristic features to achieve an 0.98 F1 score, but this takes too much processing time compared to the NWF method. Our results have higher performance than their findings (Table 6).

**Table 6:** Different researches classification method and results.

Research	Dataset	F1-score
Al-Matham & Al-Khalifa (2017)	IsPredatory publishers' database which contains 1000 + entries of predatory publishers	
Bedmuth et al. (2020)	6,268 articles from OMICS (as predatory) and 34,763 articles from BMC (as non-predatory)	**Engineering Area** Naïve Bayes:0.89 Random Forest: 0.83 Decision Tree: 0.71 **Biomedical Area** Naïve Bayes:0.96 Random Forest: 0.93 Decision: 0.90
Adnan et al. (2018)	200 websites for training 200 websites for testing (Beall’s list as predatory, directory of open access journals lists as non-predatory)	**Heuristic features** Naïve Bayes:0.95 SVM: 0.98 KNN(k = 3): 0.94 **NWF** Naïve Bayes: 0.89 SVM:0.96 KNN(k = 3): 0.93
Our results	1,636 websites for training 410 websites for testing (Predatory Journal Lists were collected from updated Beall’s and the Stop Predatory Journals lists. Legitimate journal list data were collected from the Berlin Institute of Health (BIH) Quest website)	**NWF** Gaussian Naïve Bayes: 0.89 SVM:0.952 Random Forest: 0.98 SGD:0.972 KNN(k = 4): 0.945

Our results support the validity of using feature words and *diff* scores to distinguish between legitimate and possible predatory journal websites. Our *diff* scores identified a number of words and terms that can be used to determine journal website type, a list that includes “index,” “international,” “impact,” “factor,” “peer review” and “submission,” among others. The words we identified are similar to those mentioned by Memon^51^, Rathore and Memon^34^, Cobey et al.^21^ and Berek^66^ as frequently found on predatory journal websites. The combined findings suggest that predatory websites are likely to emphasize ideas such as “peer review” and “indexing,” while legitimate journals don’t specifically mention what are considered standard aspects of the publication process. By themselves, BOW and TF-IDF classification methods and feature word sets are unlikely to completely solve the legitimate/predatory journal identification problem; additional sources of useful information include announcements from indexing organizations. For example, in 2017 the DOAJ removed journals published by the Business Perspectives company for suspected editorial misconduct (the publisher was reinstated in January 2019). AJPC also benefits from user contributions identifying predatory journals based on their personal experiences. Although it requires a time investment to verify all claims of legitimacy or deception, the accumulation of multiple reports for specific journals or publishers can improve identification accuracy. To improve the problem which start-up journals without DOAJ and Web of Science indexing could be viewed as predatory, we will consider the following factors to reflect the journal's legitimacy. For example, cooperating with the relevant professional society affiliation like the Ottawa group by Grudniewicz et al., focusing on a specific professional area and considering the realistic scope of interests reflected in journal instructions. can enhance the system's robustness.

## Conclusions

The purpose of our proposed AJPC system is to help academic authors make the best decisions for submitting their manuscripts. It is currently being used by academics in several scholarly communities in Taiwan: National Yang Ming Chiao Tung University (NYCU), the Center for Taiwan Academic Research Ethics Education, and the National Taiwan University Office of Research and Development, among others. The latest AJPC version is currently open to all interested users at https://ajpc.lib.nycu.edu.tw/. We welcome recommendations for whitelisting and blacklisting sites in order to optimize accuracy.

Research institutions and funding organizations are also interested in this issue. Universities and academic research centers could place greater emphasis on publication quality rather than quantity when assessing individuals for hiring and tenure decisions, thereby reducing the incentives for authors to consider publishing in predatory journals. Those same parties could also provide lectures and consulting services to increase awareness of predatory journal tactics. The Center for Taiwan Academic Research Ethics Education is sponsoring training activities across the country, and three organizations (DOAJ, the Committee on Publication Ethics, and the Open Access Scholarly Publishers Association) are sharing resources to launch a “Think.Check.Submit” website aimed at showing authors how to identify the best journals for their specific needs.

Regarding future plans, several changes are required to make the AJPC system more efficient—many of them minor, some requiring significant revisions. One potential problem is the blocking of web crawlers by predatory journal websites, possibly resulting in “Internal Server Error” messages causing subsequent queries to fail. Any solution to this issue must be able to handle the requirements of multiple websites. Another software-related problem has to do with journal abbreviations, which can be confusing to system users. During our tests we noticed that some journal titles that appear on the updated Beall’s and Stop Journals lists failed to appear on AJPC results screens, and we need to understand why.

Another major goal for improvement is using input from various academic resources to create a list of predatory conferences, whose proceedings are often promoted as a way to inflate researcher CVs. Lang, et al.^67^ noted that a significant lack of awareness and education about predatory journals and conferences among both medical residents and staff in the universities. In addition to the experienced teachers providing suggestions for students to publish, an effective evaluation system for conference submissions is also important. For this task, we may request permission to borrow ideas from or create links to content from the California Institute of Technology, whose library website contains a list of questionable conferences and conference organizers. There are differences between clues for predatory journals and predatory conference websites that require attention, especially the presence of for-profit sponsoring organizations located in developing countries. Some conferences emphasize their locations (e.g., holiday resorts) rather than academic or scientific exchanges while still promising listings in journal indexes such as SCI, SSCI, and EI.

## Supplementary Information

Supplementary Information 1.

Supplementary Information 2.

Supplementary Information 3.

Supplementary Information 4.

Supplementary Information 5.

Supplementary Information 6.

## Data Availability

All data generated or analyzed during this study are included in this published article and its supplementary information files. The underlying source code is available at https://github.com/nctu-dcs-lab/predatory_journals_detection.
