# Author Correction: An open automation system for predatory journal detection

**DOI:** 10.1038/s41598-023-34493-1

**Published:** 2023-05-08

**Authors:** Li‑Xian Chen, Shih‑Wen Su, Chia‑Hung Liao, Kai‑Sin Wong, Shyan‑Ming Yuan

**Affiliations:** 1 School of Big Data, Fuzhou University of International Studies and Trade, Fuzhou, 350202 China; 2 Department of Computer Science, National Yang Ming Chiao Tung University, Room 702, MIRC, No.1001, University Road, Hsinchu, 30010 Taiwan

Correction to: *Scientific Reports* https://doi.org/10.1038/s41598-023-30176-z, published online 20 February 2023

The original version of the Article contained errors. The Data Availability section was incomplete,

“All data generated or analyzed during this study are included in this published article and its supplementary information files.”

now reads:

“All data generated or analyzed during this study are included in this published article and its supplementary information files. The underlying source code is available at https://github.com/nctu-dcs-lab/predatory_journals_detection.”

Additionally, in the original version of the Supplementary Information, the legends for the Supplementary Tables were missing.

The Article and accompanying Supplementary Information file have been corrected.

