# Supplementary material for: An open automation system for predatory journal detection
Source: Sci Rep. 2023 Feb 20;13:2976. doi: 10.1038/s41598-023-30176-z (PMC9940686; doi:10.1038/s41598-023-30176-z)
Supplement: Supplementary file 6 — Supplementary Information 6. [file 41598_2023_30176_MOESM6_ESM.docx]

**Supplementary Information**

Supplementary Table S1. Contents of all journals after processing according to Figure 4.

Supplementary Table S2. The frontpage contents presented by the blacklist journal websites.

Supplementary Table S3. The webpage contents of the whitelist after processing according to Figure 4.

Supplementary Table S4. The webpage contents of the blacklist after processing according to Figure 4.

Supplementary Table S5. The frontpage contents presented by the whitelist journal websites.
